# The Prognostic Impact of Kidney Dysfunction in Unselected Patients Undergoing Coronary Angiography: In What Subgroups Does Kidney Dysfunction Matter?

**DOI:** 10.3390/jcm14113753

**Published:** 2025-05-27

**Authors:** Philipp Steinke, Ibrahim Akin, Lasse Kuhn, Thomas Bertsch, Kathrin Weidner, Mohammad Abumayyaleh, Jonas Dudda, Jonas Rusnak, Mahboubeh Jannesari, Fabian Siegel, Christel Weiß, Daniel Duerschmied, Michael Behnes, Tobias Schupp

**Affiliations:** 1 Department of Cardiology, Angiology, Haemostaseology and Medical Intensive Care, University Medical Centre Mannheim, Medical Faculty Mannheim, University of Heidelberg, 68167 Mannheim, Germany; philipp.steinke@stud.uni-heidelberg.de (P.S.); ibrahim.akin@umm.de (I.A.); lasse.kuhn@stud.uni-heidelberg.de (L.K.); kathrin.weidner@umm.de (K.W.); mohammad.abumayyaleh@umm.de (M.A.); jonas.dudda@umm.de (J.D.); daniel.duerschmied@umm.de (D.D.); michael.behnes@umm.de (M.B.); 2 Institute of Clinical Chemistry, Laboratory Medicine and Transfusion Medicine, Nuremberg General Hospital, Paracelsus Medical University, 90419 Nuremberg, Germany; thomas.bertsch@klinikum-nuernberg.de; 3 Department of Cardiology, Angiology and Pneumology, University Hospital Heidelberg, 69120 Heidelberg, Germany; jonas.rusnak@med.uni-heidelberg.de; 4 Department of Biomedical Informatics, Center for Preventive Medicine and Digital Health (CPD), Medical Faculty Mannheim, Heidelberg University, 68167 Mannheim, Germany; mahboubeh.jannesari@medma.uni-heidelberg.de (M.J.); fabian.siegel@medma.uni-heidelberg.de (F.S.); 5 Department of Biomathematics and Medical Statistics, University Medical Centre Mannheim, Medical Faculty Mannheim, University of Heidelberg, 68167 Mannheim, Germany; christel.weiss@medma.uni-heidelberg.de

**Keywords:** coronary angiography, coronary artery disease, renal dysfunction, eGFR, prognosis

## Abstract

**Background/Objectives**: In recent decades, shifting demographics and advancements in treating cardiovascular disease have altered the types of patients receiving coronary angiography (CA). However, data investigating the impact of kidney dysfunction stratified by the indication for CA are limited. **Methods**: Consecutive patients who underwent invasive CA at one institution between 2016 and 2022 were included in this study. Firstly, the prevalence and extent of coronary artery disease (CAD) in patients with different levels of kidney function was assessed. Secondly, the study examined how impaired kidney function affected long-term outcomes—specifically the risk of rehospitalization for heart failure (HF), acute myocardial infarction (AMI), or the need for coronary revascularization—at 36 months of follow-up. **Results**: A total of 7624 patients undergoing CA were included with a median estimated glomerular filtration rate (eGFR) of 68.9 mL/min/1.73 m^2^ (IQR: 50.8–84.3). In total, 63.7% of patients had an eGFR ≥ 60 mL/min/1.73 m^2^, 29.0% an eGFR of 30–<60 mL/min/1.73 m^2^, and 7.3% an eGFR of <30 mL/min/1.73 m^2^. Compared to patients with an eGFR ≥ 60 mL/min/1.73 m^2^, those with an eGFR 30–<60 mL/min/1.73 m^2^ and eGFR < 30 mL/min/1.73 m^2^ had a higher prevalence of CAD (66.8% vs. 72.9% and 80.1%, respectively; *p* = 0.001) and three-vessel CAD (25.6% vs. 34.5% and 39.5%, respectively; *p* = 0.001). At 36 months of follow-up, patients with an eGFR 30–<60 mL/min/1.73 m^2^ and eGFR < 30 mL/min/1.73 m^2^ suffered from significantly higher risk of HF-associated rehospitalization (HR = 1.937, 95% CI: 1.739–2.157, *p* = 0.001 and HR = 3.223, 95% CI: 2.743–3.787, *p* = 0.001, respectively) and AMI compared to patients with an eGFR ≥ 60 mL/min/1.73 m^2^ (reference group). The significantly higher risk of HF-related rehospitalization remained after multivariable adjustment. **Conclusions**: Both groups with impaired kidney function demonstrated a markedly higher risk of rehospitalization for HF at 36 months—even after multivariate adjustments. Increased risk of HF-related rehospitalization in patients with an eGFR < 30 mL/min/1.73 m^2^ was especially evident if they also presented with decompensated HF and LVEF < 35%. In patients with an eGFR 30–<60 mL/min/1.73 m^2^, presenting with angina pectoris and multivessel disease increased the risk of HF-related rehospitalization.

## 1. Introduction

Coronary artery disease (CAD) is the primary cause of mortality globally [1]. Patients with CAD frequently suffer from comorbidities, which play a significant role in shaping prognosis and clinical outcomes [2,3]. Kidney disease is one of these common comorbidities with a prevalence of up to 29% in CAD patients [4]. Moreover, it represents a major risk factor for CAD [5,6,7]. Despite vast improvements in treatment strategies over the past decades, kidney dysfunction has recently been shown to independently predict adverse outcomes in patients with CAD, arrythmias, and HF [6,8].

Patients with impaired kidney function have been found to exhibit common “traditional” CAD risk factors such as old age, hypertension, diabetes, dyslipidemia, and physical inactivity [6,9,10,11]. More directly, renal failure is a vasculopathic state [10]. Other “non-traditional” cardiovascular disease factors including sympathetic nervous system activation, inflammation, elevated oxidized low-density lipoprotein levels [6,12,13,14,15], endothelial dysfunction and renal anemia [6,16] are all associated with renal disease and act in an atherogenic manner, serving to disrupt myocardial microcirculation and accelerate atherosclerotic plaque formation and rupture [10,17]. Studies have found that, as the estimated glomerular filtration rate (eGFR) declines, CAD is more prevalent, severe, progresses more rapidly and clinical manifestations of CAD worsen [6,17,18,19].

The occurrence and prognostic value of kidney dysfunction in unselected patients receiving coronary angiography (CA) has not been thoroughly explored. This, together with the fact that humanity is experiencing substantial ongoing demographic changes and that cardiovascular treatment strategies have immensely improved over the last decades, highlights the need for the accurate characterization of individuals undergoing CA.

Consequently, this study, using a large retrospective registry-based dataset, sought to investigate the prognostic impact of varying degrees of kidney function, in a cohort of unselected patients undergoing CA.

## 2. Materials and Methods

### 2.1. Study Patients, Design, and Data Collection

This study included all consecutive patients who underwent CA at the University Medical Centre Mannheim (UMM), Germany, from January 2016 to August 2022. Patient identification was carried out using Operation and Procedure Classification (OPS) codes. Relevant clinical data related to the index event—including presenting symptoms, initial diagnoses, medical background, angiographic findings, interventions, and discharge medications—were retrospectively gathered using the in-house electronic hospital information system (SAP^®^, Walldorf, Germany). All patients were included only once, irrespective of the number of CA they received. The UMM operates a general emergency department handling surgical, traumatic, cardiovascular, and neurological emergencies. The cardiology department is equipped with a 24-h catheterization lab, an electrophysiology lab, a hybrid operating room, and telemetry units. Additionally, the UMM is part of a well-established grid of medical centers providing cardiac surgeries such as coronary artery bypass grafting (CABG) for referred patients.

This study’s patient collective was drawn from a retrospective, single-center registry comprising consecutive patients receiving CA during hospitalization at the UMM (DRKS–ID: DRKS00032897). This registry was established in accordance with the ethical standards outlined in the Declaration of Helsinki and received approval from the Medical Ethics Committee II of the Medical Faculty Mannheim, University of Heidelberg, Germany (ethical approval code: 829-22).

### 2.2. Inclusion and Exclusion Criteria

This study included all consecutive patients undergoing invasive CA between January 2016 and August 2022 at our institution with at least 18 years of age. In-house interventional cardiologists performed all CAs in accordance with European guidelines [20]. For the present study, two independent cardiologists reassessed all source data of CA examinations (such as imaging files) and reports. For the present study, patients under the age of 18 and patients without the necessary data to calculate eGFR were excluded. All eGFRs were calculated using the Chronic Kidney Disease (CKD) Epidemiology collaboration equation. This equation has been shown to be accurate in patients with CAD [21]. This study did not implement any further exclusion criteria other than those mentioned above.

### 2.3. Risk Stratification

Patients were allocated into the following three kidney function groups: eGFR < 30 mL/min/1.73 m^2^, eGFR 30–<60 mL/min/1.73 m^2^, and eGFR ≥ 60 mL/min/1.73 m^2^. If patients had multiple eGFR measurements, risk stratification was performed according to the median eGFR. Additional risk stratification was performed, stratifying eGFR further into the following subgroups: ≥90 mL/min/1.73 m^2^, 60–89 mL/min/1.73 m^2^, 45–59 mL/min/1.73 m^2^, 30–44 mL/min/1.73 m^2^, 15–29, <15 mL/min/1.73 m^2^). Moreover, the prognostic impact of eGFR as a continuous variable (i.e., per 1 mL/min/1.73 m^2^ increase) was investigated.

### 2.4. Study Endpoints

The primary endpoint investigated in this study was rehospitalization for HF at 36 months. The secondary endpoints were acute myocardial infarction (AMI) and coronary revascularization, both within 36 months. International Classification of Diseases (ICD) codes at the UMM, Germany, were used to define all endpoints.

### 2.5. Statistical Methods

Quantitative data are presented as the mean ± standard error of the mean (SEM), the median and the interquartile range (IQR), or as ranges, depending on the associated distributions. For data with a normal distribution, comparisons were made using the Student’s t-test, whereas the Mann–Whitney U test was applied to data not following a normal distribution. For the assessment of deviations from a Gaussian distribution, the Kolmogorov–Smirnov test was used. Absolute and relative frequencies are used to illustrate qualitative data. As applicable, either the Chi-square test or Fisher’s exact test was utilized in the comparison of qualitative data. Kaplan–Meier (KM) analyses were executed while investigating the risk of the three endpoints of this study within different eGFR groups—univariable hazard ratios (HR) were presented with 95% confidence intervals. This was also carried out for further eGFR risk stratification. The prognostic impact of kidney dysfunction was investigated through multivariable Cox regression models. The “forward selection” option was selected for this step. For the selection of variables for the multivariable model, a univariate *p*-value threshold of *p* < 0.1 was used. Additionally, the proportional hazards assumption was investigated using weighted Schoenfeld residuals using the SAS (Version 9.4, SAS Institute Inc., Cary, North Carolina, USA) option ZPH for each of the following variables: sex, diabetes, CAD, myocardial infarction (MI), CABG, ST-elevation myocardial infarction (STEMI), atrial fibrillation (AF), decompensated HF, and eGFR groups. The prognostic impact of eGFR increases per 1 mL/min/1.73 m^2^ was also investigated using a multivariable cox regression model. Multivariable Cox regression analyses were also performed for pre-specified subgroups: age ≥ 75 and age < 75, the existence of unstable angina (AP), (non-) ST elevation myocardial infarction (N)STEMI and acute decompensated heart failure (ADHF) at admission to hospital, multivessel disease, and left ventricular ejection fraction (LVEF). Forest plots were used to illustrate multivariable Cox regression analyses.

Results were considered significantly if statistical tests issued *p* ≤ 0.05. The statistics program SPSS (Version 25, IBM, Armonk, NY, USA) was used for all statistical analyses bar the proportional hazards assumption proof.

## 3. Results

### 3.1. Study Population

From January 2016 to August 2022, CA was performed in 7691 individuals at the catheterization unit of the University Medical Centre Mannheim (UMM), Germany. After excluding 67 patients due to missing eGFR data, the study cohort comprised 7624 patients. The median eGFR of this cohort was 68.9 mL/min/1.73 m^2^ (IQR: 50.8–84.3 mL/min/1.73 m^2^). Of these 7624 patients, 7.28% (*n* = 554) had an eGFR of <30 mL/min/1.73 m^2^, 29.06% (*n* = 2213) an eGFR of 30–<60 mL/min/1.73 m^2^, and 63.79% (*n* = 4857) an eGFR of ≥60 mL/min/1.73 m^2^.

Patients’ characteristics and comorbidities are outlined in Table 1. Patients with an eGFR < 30 mL/min/1.73 m^2^ and eGFR 30–<60 mL/min/1.73 m^2^ were of advanced age (median age 76 and 77 vs. 65 years; *p* = 0.001) and possessed a higher body mass index (BMI) (median BMI: 27.8 and 27.7 vs. 27.2; *p* = 0.002) compared to patients with an eGFR ≥ 60 mL/min/1.73 m^2^ (Table 1). Patients with an eGFR ≥ 60 mL/min/1.73 m^2^ were most commonly males (69.8%), followed by those with an eGFR < 30 mL/min/1.73 m^2^ (60.1%) and an eGFR 30–<60 mL/min/1.73 m^2^ (55%) (*p* = 0.001). Regarding cardiovascular risk factors, patients with an eGFR < 30 mL/min/1.73 m^2^ and eGFR 30–<60 mL/min/1.73 m^2^ displayed higher prevalences of diabetes (43.0% and 39.0% vs. 24.6%, *p* = 0.001), but lower prevalences of hyperlipidemia (28.2% and 31.4% vs. 38.3%, *p* = 0.001) than those with an eGFR > 60 mL/min/1.73 m^2^. The cohort of patients with an eGFR 30–<60 mL/min/1.73 m^2^ had the highest rate of arterial hypertension (86.5%), followed by the eGFR ≥ 60 mL/min/1.73 m^2^ (85.4%) and eGFR < 30 mL/min/1.73 m^2^ (78.7%) cohorts (*p* = 0.001). Moreover, patients with an eGFR < 30 mL/min/1.73 m^2^ and eGFR 30–<60 mL/min/1.73 m^2^ had higher rates of pre-existing congestive HF (21.7% and 13.7% vs. 5.1%, *p* = 0.001) and pre-existing stroke (1.2% and 0.6% vs. 0.5%, *p* = 0.035), in comparison to patients with an eGFR ≥ 60 mL/min/1.73 m^2^ (Table 1). Significantly higher rates of moderately reduced LVEF (35–44%: 18.6% and 16.8% vs. 12.3%, *p* = 0.001) and markedly reduced LVEF (<35%: 29.4% and 21.0% vs. 0.8%, *p* = 0.001) were found in patients with an eGFR < 30 mL/min/1.73 m^2^ and eGFR 30–<60 mL/min/1.73 m^2^ compared to those with an eGFR ≥ 60 mL/min/1.73 m^2^ (Table 1).

Accordingly, CAD was found more frequently in patients with an eGFR < 30 mL/min/1.73 m^2^ and eGFR 30–<60 mL/min/1.73 m^2^ (no evidence of CAD: 19.9% and 27.1% vs. 33.2%, *p* = 0.001). In line, more severe CAD was also found amongst patients exhibiting an eGFR < 30 mL/min/1.73 m^2^ and eGFR 30–<60 mL/min/1.73 m^2^, (three-vessel disease: 39.5% and 34.5% vs. 25.6%, *p* = 0.001) (Table 2). As a result, they received CABG (1.8% and 1.1% vs. 0.4%, *p* = 0.001) more often than the patients exhibiting an eGFR ≥ 60 mL/min/1.73 m^2^. Regarding laboratory data, the patients with an eGFR < 30 mL/min/1.73 m^2^ and eGFR 30–<60 mL/min/1.73 m^2^ had higher levels of NT-pro BNP (median 11,261 and 3287 vs. 1168 pg/mL; *p* = 0.001) compared to patients exhibiting an eGFR ≥ 60 mL/min/1.73 m^2^ (Table 2).

### 3.2. Prognostic Value of Reduced Kidney Function in Patients Undergoing CA

**Figure 1:**
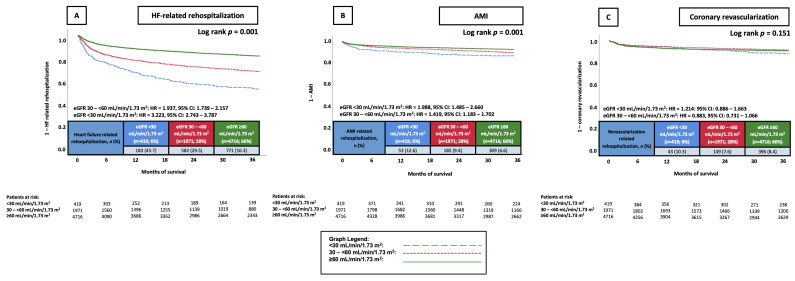
KM curves illustrating the prognostic impact of different eGFR groups on the risk of rehospitalization due to HF (**A**), AMI (**B**), and coronary revascularization (**C**), all at 36 months. AMI, acute myocardial infarction; CI, confidence interval; eGFR, estimated glomerular filtration rate; HF, heart failure; HR, hazard ratio; KM, Kaplan–Meier.

Patients who had an eGFR < 30 mL/min/1.73 m^2^ and eGFR 30–<60 mL/min/1.73 m^2^ had significantly higher risk of rehospitalization due to worsening HF at 36 months (43.7% and 29.5% vs. 16.3%; eGFR < 30 mL/min/1.73 m^2^: HR = 3.223, 95% CI: 2.743–3.787, *p* = 0.001, eGFR 30–<60 mL/min/1.73 m^2^: HR = 1.937, 95% CI: 1.739–2.157, *p* = 0.001) and rehospitalization due to AMI at 36 months (12.6% and 9.4% vs. 6.6%; eGFR < 30 mL/min/1.73 m^2^: HR = 1.988, 95% CI: 1.485–2.660, *p* = 0.001, eGFR 30–<60 mL/min/1.73 m^2^: HR = 1.419, 95% CI: 1.183–1.702, *p* = 0.001) compared to patients with an eGFR ≥ 60 mL/min/1.73 m^2^. While patients exhibiting an eGFR of <15 mL/min/1.73 m^2^ did have higher revascularization rates than patients within the other two eGFR groups, this difference was not statistically significant (10.3% vs. 7.6% and 8.4; *p* = 0.165). Patients exhibiting an eGFR 30–<60 mL/min/1.73 m^2^ also did not have a significantly different risk to the reference group (HR = 0.883, 95% CI: 0.731–1.066, *p* = 0.195) (Figure 1A–C).

**Figure 2:**
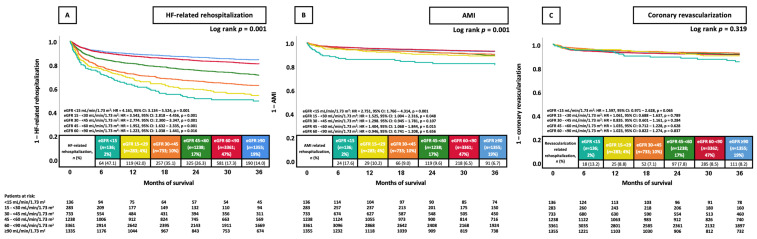
KM curves illustrating the prognostic impact of further stratified eGFR groups on the risk of HF-related rehospitalization (**A**), AMI (**B**), and coronary revascularization (**C**), all at 36 months. AMI, acute myocardial infarction; CI, confidence interval; eGFR, estimated glomerular filtration rate; HF, heart failure; HR, hazard ratio; KM, Kaplan–Meier.

When stratifying eGFR further (i.e., ≥90 mL/min/1.73 m^2^, 60–89 mL/min/1.73 m^2^, 45–59 mL/min/1.73 m^2^, 30–44 mL/min/1.73 m^2^, 15–29, <15 mL/min/1.73 m^2^), patients with lower eGFR categories were linked to a significantly higher risk of rehospitalization due to HF at 36 months when compared to the eGFR ≥ 90 mL/min/1.73 m^2^ group (all *p* < 0.005, Figure 2A). In contrast, only patients with an eGFR of <15 mL/min/1.73 m^2^, 15–<30 mL/min/1.73 m^2^, and 45–<60 mL/min/1.73 m^2^ exhibited a significantly elevated AMI risk at 36 months in contrast to patients who had an eGFR ≥ 90 mL/min/1.73 m^2^ (Figure 2B). However, the risk of coronary revascularization was not affected by eGFR (all *p* > 0.05, Figure 2C).

### 3.3. Multivariable Cox Regression Analyses

The proportional hazards assumption held for all investigated variables bar CAD (*p* = 0.0262) and STEMI (*p* = 0.0092). Upon analysis of the associated Kaplan–Meier curves, however, it was evident that the overlap of the curves causing the violation of the proportional hazard assumption was only at the very beginning of the observation time. When performing the same analysis but excluding the first 5 months, the proportional hazards assumptions were no longer violated (CAD: *p* = 0.2131 and STEMI: *p* = 0.0881).

**Figure 3:**
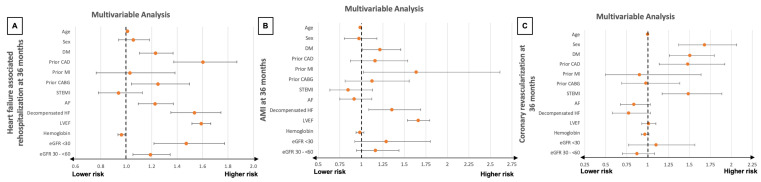
Forest plots illustrating the results of multivariable Cox regression analyses with regard to the risk of HF-related rehospitalization (**A**), AMI (**B**), and coronary revascularization (**C**), all at 36 months within the entire study cohort. AF, atrial fibrillation; AMI, acute myocardial infarction; CABG, coronary artery bypass grafting; CAD, coronary artery disease; eGFR, estimated glomerular filtration rate; HF, heart failure; LVEF, left ventricular ejection fraction; STEMI, ST elevation myocardial infarction.

Even after multivariable adjustments, an eGFR < 30 mL/min/1.73 m^2^ and eGFR 30–<60 mL/min/1.73 m^2^ were still associated with a significant increase in risk of rehospitalization for HF at 36 months (eGFR < 30 mL/min/1.73 m^2^: HR = 1.470, 95% CI: 1.218–1.774; *p* = 0.001, eGFR 30–<60 mL/min/1.73 m^2^: HR = 1.189, 95% CI: 1.052–1.345, *p* = 0.006) (Figure 3A). Additionally, advanced age (HR = 1.009, 95% CI: 1.004–1.014; *p* = 0.001), the presence of diabetes mellitus (HR = 1.229, 95% CI: 1.104–1.369; *p* = 0.001), prior CAD (HR = 1.603, 95% CI: 1.373–1.871; *p* = 0.001), prior CABG (HR = 1.249, 95% CI: 1.042–1.497; *p* = 0.016), AF (HR = 1.225, 95% CI: 1.095–1.371; *p* = 0.001), ADHF (HR = 1.535, 95% CI: 1.351–1.744; *p* = 0.001), and worse LVEF (HR = 1.589, 95% CI: 1.516–1.665; *p* = 0.001) were all linked to an increased likelihood of rehospitalization due to HF within 36 months (Figure 3A). On the other hand, a higher median hemoglobin was linked to a lower risk (HR = 0.961, 95% CI: 0.935–0.989; *p* = 0.006) (Figure 3A).

After multivariate adjustment, eGFR was no longer linked to significantly higher risk of AMI (eGFR < 30 mL/min/1.73 m^2^: HR = 1.289, 95% CI: 0.923–1.800; *p* = 0.137, eGFR = 30–<60 mL/min/1.73 m^2^: HR = 1.164, 95% CI: 0.942–1.437, *p* = 0.159) (Figure 3B).

Moreover, even when analyzing eGFR as a continuous variable (i.e., per 1 mL/min increases), higher eGFR values were associated with a lower risk of HF-related rehospitalization at 36 months (HR = 0.994, 95% CI: 0.992–0.997, *p* = 0.001). No significant difference was found for AMI at 36 months (HR = 0.999, 95% CI: 0.995–1.003, *p* = 0.618) or coronary revascularization at 36 months (HR = 1.001, 95% CI: 0.998–1.005, *p* = 0.534) when analyzing the prognostic impact of eGFR as a continuous variable.

### 3.4. Prognostic Impact of Kidney Dysfunction in Pre-Specified Subgroups

**Figure 4:**
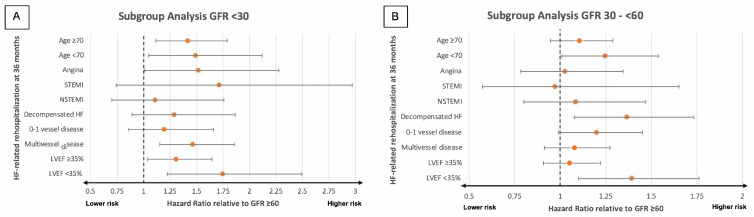
Forest plots illustrating the results of subgroup analyses investigating the prognostic impact of different levels of eGFR regarding the likelihood of rehospitalization due to HF within 36 months in patients with eGFR < 30 mL/min/1.73 m^2^ (**A**) and eGFR 30–<60 mL/min/1.73 m^2^ (**B**). GFR, glomerular filtration rate; HF, heart failure; LVEF, left ventricular ejection fraction; N(STEMI), non-ST elevation myocardial infarction.

An eGFR < 30 mL/min/1.73 m^2^ was linked to with a significantly elevated risk of rehospitalization due to HF at 36 months in patients presenting with AP (HR = 1.518, 95% CI: 1.012–2.276; *p* = 0.044), multivessel disease (HR = 1.465, 95% CI: 1.152–1.861; *p* = 0.002), and both LVEF ≥35% (HF = 1.307, 95% CI: 1.037–1.645; *p* = 0.023) and <35% (HR = 1.746, 95% CI: 1.222–2.494; *p* = 0.002) at index hospitalization compared to patients exhibiting an eGFR ≥60 mL/min/1.73 m^2^ (Figure 4A). There was no statistically significant increase in the risk of rehospitalization due to HF among patients who presented with STEMI (HR = 1.714, 95% CI: 0.740–2.971; *p* = 0.208), NSTEMI (HR = 1.109, 95% CI: 0.699–1.759; *p* = 0.661), decompensated HF (HR = 1.288, 95% CI: 0.890–1.865; *p* = 1.180), or single/no vessel disease (HR = 1.194, 95% CI: 0.858–1.662; *p* = 0.292) (Figure 4A).

Patients with an eGFR 30–<60 mL/min/1.73 m^2^ faced a notably elevated risk of rehospitalization due to HF at 36 months if they also presented with LVEF < 35% (HR = 1.392, 95% CI: 1.100–1.762, *p* = 0.006), but not LVEF ≥35% (HR = 1.051, 95% CI: 0.907–1.219; *p* = 0.507), AP (HR = 1.026, 95% CI: 0.783–1.345; *p* = 0.854), or multivessel disease (HR = 1.078, 95% CI: 0.913–1.273; *p* = 0.376) (Figure 4B). Moreover, an eGFR 30–<60 mL/min/1.73 m^2^ did not correspond to a significantly elevated risk of rehospitalization due to HF if also presenting with STEMI (HR = 0.972, 95% CI: 0.572–1.652; *p* = 0.917), NSTEMI (HR = 1.084, 95% CI: 0.800–1.469; *p* = 0.603), and single/no vessel disease (HR = 1.200, 95% CI: 0.990–1.453; *p* = 0.063). An eGFR 30–<60 mL/min/1.73 m^2^ was, however, associated with a significantly higher risk in patients presenting with decompensated HF (HR = 1.366, 95% CI: 1.077–1.733; *p* = 0.010) (Figure 4B).

## 4. Discussion

This study investigated the prevalence and prognostic value of the presence and degree of kidney dysfunction in a large cohort of unselected patients undergoing CA, recruited consecutively, without selection criteria, between January 2016 and August 2022. From a total of 7624 patients undergoing CA, 63.7% exhibited an eGFR ≥60 mL/min/1.73 m^2^, 29.0% presented with an eGFR 30–<60 mL/min/1.73 m^2^), and 7.3% had an eGFR < 30 mL/min/1.73 m^2^. An eGFR < 30 mL/min/1.73 m^2^ and eGFR 30–<60 mL/min/1.73 m^2^ were associated with significantly higher HF- and AMI-related rehospitalization at 36 months. This relationship (i.e., a lower eGFR is associated with a higher risk of a given endpoint) was also demonstrated when further stratifying eGFR groups, but only for HF-related rehospitalization. A higher risk of HF-related rehospitalization in patients with an eGFR < 30 mL/min/1.73 m^2^ and eGFR 30–<60 mL/min/1.73 m^2^ was confirmed after multivariable adjustment even when including eGFR as a continuous variable. The prognostic impact of having an eGFR < 30 mL/min/1.73 m^2^ was particularly noticeable in patients who exhibited multivessel disease, AP, and those with both an LVEF < 35% and an LVEF ≥35%. In contrast, individuals with an eGFR 30–<60 mL/min/1.73 m^2^ faced a notably increased risk of rehospitalization due to HF when presenting with cardiac decompensation and an LVEF < 35%.

Prevalences of kidney dysfunction in patients with CAD range from 18% to 29% [4,22] and thus represent one of the most common non-cardiac comorbidities in CAD patients. Despite this, studies investigating the prognostic value of kidney dysfunction in consecutive, unselected patients remain sparse. The prognostic impact of kidney dysfunction identified in this study is not dependent on a specific diagnostic entity. This is due to the fact that this study includes all consecutive patients undergoing CA, without the application of any additional exclusion criteria. This approach allows for a broad comparison of the prognostic implications of kidney dysfunction, both irrespective of underlying diagnoses and across various patient subgroups. Consequently, this study provides a valuable representation of the contemporary patient population, which—as a result of current demographic shifts—is burdened more and more by both cardiac and non-cardiac comorbidities, thereby complicating risk assessment.

As mentioned previously, kidney dysfunction is associated with both traditional cardiovascular disease (CVD) risk factors, such as diabetes mellitus and hypertension, and non-traditional, uremia-related factors including oxidative stress, inflammation, endothelial dysfunction, and the accumulation of uremic toxins [6,11]. In the present study, patients with a lower kidney function (eGFR < 30 mL/min/1.73 m^2^ and 30–<60 mL/min/1.73 m^2^) were accompanied by significantly higher rates of diabetes than our reference group (eGFR ≥ 60 mL/min/1.73 m^2^). While traditional risk factors are primarily responsible for the initiation of atherosclerosis in the early stages of kidney disease, evidence suggests that non-traditional risk factors play a progressively dominant role as renal function declines [6,23,24]. Lipoprotein modification—particularly the oxidation of low-density lipoproteins—alongside higher levels of uremic toxins including indoxyl sulfate and p-cresyl sulfate adds to endothelial dysfunction and vascular smooth muscle cell proliferation, thereby accelerating the development and progression of CAD [6,25,26,27,28,29,30]. Moreover, asymmetric dimethylarginine, an endogenous competitive inhibitor of endothelial nitric oxide synthase, is often higher in kidney disease patients and impairs nitric oxide bioavailability, exacerbating vascular dysfunction [31]. Oxidative stress and chronic inflammation further promote coronary microcalcification, a pathological feature associated with ACS, due to its detrimental effects on myocardial microcirculation [27,28,29,30,32]. Collectively, these interrelated mechanisms likely contribute to a greater incidence of major adverse cardiovascular events, including HF and AMI, in individuals with kidney dysfunction [33].

When investigating the prognostic impact of kidney dysfunction, many studies focus on all-cause mortality as the primary endpoint. In a recent study that investigated the interaction between three-vessel CAD and low eGFR (<60 mL/min/1.73 m^2^) in a cohort of 1017 consecutive subjects undergoing CA, Piscitelli et al. found low eGFR to significantly amplify the already significantly higher risk of all-cause mortality associated with three-vessel disease [34]. The same authors had also previously found a strong association between kidney disease measures (e.g., reduced GFR and albuminuria) and coronary atherosclerotic burden [35]. This relationship is so significant that both the authors and the American Heart Foundation have labeled kidney disease as a CAD risk equivalent for cardiovascular endpoints [34,36]. Additionally, while investigating a cohort of 23,178 CAD patients undergoing CA, Chen et al. not only found severe kidney dysfunction (eGFR < 30 mL/min/1.73 m^2^) to be linked to a significantly elevated risk of all-cause mortality risk than moderate dysfunction (eGFR 30–60 mL/min/1.73 m^2^), but they also discovered a U-shaped relationship between eGFR and mortality [22]. At an eGFR of above 100 mL/min/1.73 m^2^, all-cause mortality started to increase. This U-shaped relationship is in line with previous studies [37,38,39,40,41,42]. Even when adjusting for muscle mass, a variable potentially impeding eGFR calculations, hyperfiltration was still found to increase the risk of all-cause mortality [43]. Glomerular hyperfiltration has been reported to increase the risk of coronary artery calcification in CAD patients, while a high GFR has also been associated with mortality risk increasing factors such as total carotid plaque area and left ventricular hypertrophy [44,45]. As such, the interaction between hyperfiltration and CAD may explain the aforementioned U-shaped relationship between eGFR and mortality.

The prognostic role of in-hospital bleedings in patients admitted for ACS has also recently been investigated. Spadafora et al., through the investigation of 23,270 ACS patients enrolled in the PRAISE registry, showed all-cause mortality, reinfarction, and major bleeding to be significantly higher at the 1-year follow-up in the patient group suffering in-hospital bleeding [46].

Several authors demonstrate that kidney dysfunction may be linked to a significantly higher risk of HF-related hospitalization [47,48,49]. Furthermore, and in accordance with our results, late-stage kidney disease (eGFR < 60 mL/min/1.73 m^2^) has been associated with higher rates of HF-related hospitalization compared to patients with early-stage kidney dysfunction patients (GFR ≥ 60 mL/min/1.73 m^2^) [47,48]. Regarding HF subgroup analysis, however, the data remains inconclusive. Hillege et al. investigated the prognostic value of renal function in patients with chronic HF using data from the CHARM study and found that renal dysfunction (eGFR < 60 mL/min/1.73 m^2^) was independently associated with an elevated risk of mortality and HF-related hospitalization for both patients with HF with a reduced ejection fraction (HFrEF) and HF with a preserved ejection fraction (HFpEF) patients [49]. Our study’s subgroup analysis confirms this result only for patients with an eGFR < 30 mL/min/1.73 m^2^ in a cohort of unselected patients. An eGFR 30–<60 mL/min/1.73 m^2^ was linked to an elevated risk of rehospitalization due to HF only in individuals suffering from an LVEF < 35% (approximately HFrEF), not those with an LVEF ≥ 35% (approximately HFmrEF and HFpEF).

In contrast to the findings of Hillege et al., a recent study including 1932 acute HF patients investigated the prognostic impact of renal function in HFpEF and HFrEF patients and found that kidney dysfunction (eGFR < 60 mL/min/1.73 m^2^) was only a significant prognostic marker for readmission in patients with HFrEF, not HFpEF [48]. The authors, Park et al., also stratified according to severity of renal dysfunction and found that both moderate (30–<60 mL/min/1.73 m^2^) and severe (GFR < 30 mL/min/1.73 m^2^) kidney dysfunction were associated with significantly higher rates of readmission, but only for patients with HFrEF [48]. Additionally, the same authors found that, after multivariate adjustment, only HFrEF patients with severe kidney function were subject to a higher risk of 12-month mortality in contrast to patients with HFpEF or moderate kidney dysfunction. Similarly, the MAGGIC meta-analysis which included 20,754 HF patients found both moderate and severe kidney function to only be a predictor for all-cause mortality in HFrEF patients [50].

Conversely, Ahmed et al., conducting a post-hoc propensity score matched analysis of the Digoxin Investigation Group trial, a study including 7788 ambulatory patients with chronic HF, were one of the first to observe significantly higher kidney dysfunction associated mortality rates in patients with diastolic HF (HFpEF) than in those with systolic HF (HFrEF) [51]. Since then, this phenomenon has been corroborated by several authors including a meta-analysis by Damman et al. [52]. The authors postulate that the reason kidney dysfunction is associated with a higher risk of mortality in patients with HFpEF include the same traditional risk factors shared between kidney dysfunction and CAD, such as hypertension and diabetes. These risk factors are associated both with impaired eGFR and worse outcomes. Generally, patients with HFpEF possess differing clinical and biochemical profiles, resulting in many potential reasons for the observed effect. [52].

Regarding HF with mildly reduced ejection fraction (HFmrEF), a recent study has investigated the prognostic impact of the severity and etiology of CKD in 2155 patients with HFmrEF [53]. This study found that even milder stages of CKD (i.e., Kidney Disease Improving Global Outcomes (KDIGO) stage 3a) were associated with an increased all-cause mortality risk at 30 months. Interestingly, the highest risk of rehospitalization due to HF was found in patients with KDIGO stage 3b and 4, not KDIGO stage 5 [53]. In contrast to this, the etiology of CKD was not shown to be associated with either the 30-month all-cause mortality risk or the risk of rehospitalization due to HF.

The prognostic impact of different kidney function levels on AMI patients has been thoroughly investigated for mortality as well as HF hospitalization [54,55,56,57]. When considering AMI subgroups, NSTEMI and STEMI cohorts are often investigated separately; the comparison of both is scarce. The majority of authors find kidney dysfunction (eGFR < 60 mL/min/1.73 m^2^) to be associated with a higher risk of mortality for both STEMI [58,59,60,61] and NSTEMI [62,63,64,65] cohorts whether stratifying kidney dysfunction, or not. This is also the case for HF associated rehospitalization [66]. Another interesting and highly relevant phenomenon in AMI patients undergoing PCI—specifically diabetic patients with AMI—is the impact of SGLT2-inhibitors on acute kidney injury (AKI) caused by contrast. A recent multicenter international study by Paolisso et al., using the data of 636 patients from the SGLT2-I AMI PROTECT registry, found that in type 2 diabetes mellitus patients with AMI undergoing PCI, usage of SGLT2-I was linked to a nephroprotective effect [67]. Moreover, concomitant cardiogenic shock (CS) has been recently shown to significantly interact with AKI regarding clinical endpoints [68]. In this study, the investigation of 219 CS patients showed that early AKI affects more than half of CS patients and is linked to higher all-cause mortality at 30 days in CS patients [68]. The aforementioned studies clearly indicate that kidney dysfunction remains s highly relevant and pressing issue that warrants further investigation. 

### Study Limitations

The study possesses several constraints. Owing to its retrospective nature and single-center design, the findings presented in this manuscript could be susceptible to both measured and unmeasured confounding variables. Due to the use of Operation and Procedure Classification (OPS) codes, lower documented event rates of prior medical conditions may occur. One of the primary limitations of this study was the absence of post-discharge data on all-cause mortality, which is particularly important considering the potential interplay between morality and rehospitalization. Despite this, however, kidney dysfunction appeared to be an independent risk factor indicating the relevance and importance of rehospitalization as an endpoint. Another limitation is assessment of all endpoints at our institution only. Moreover, due to a lack of data, there was no stratification into acute kidney failure and chronic kidney disease and no inclusion of previous eGFR data to investigate kidney function dynamics. Furthermore, there was no separate analysis conducted on patients receiving dialysis. Similarly, the investigation of cardiovascular comorbidities at index hospitalization did not include chronic coronary syndrome as an investigated medical entity and thus this was not investigated as a possible indication for CA. Additionally, due to the fact that severely reduced kidney function may pose as a contraindication for coronary angiography, some patients with severely reduced kidney function may not have been included in this registry. Finally, the prognostic impact of SGLT2 inhibitors, which are both reno- and cardioprotective, was not able to be investigated due to the low prescription rate during the investigated time period.

## 5. Conclusions

Kidney dysfunction, with a recorded prevalence of 36.3%, represented a very common comorbidity in unselected patients undergoing CA. Both a kidney function of < 30 mL/min/1.73 m^2^ and 30–<60 mL/min/1.73 m^2^ was linked to a significantly elevated risk of rehospitalization due to HF at 36 months—this relationship also persisted after multivariable adjustment. Additionally, patients presenting with AP and multivessel disease were especially susceptible to HF-related rehospitalization if they had an eGFR of <30 mL/min/1.73 m^2^, while an eGFR of 30–<60 mL/min/1.73 m^2^ was related to a significantly heightened risk of being rehospitalized due to HF in patients suffering from decompensation HF and LVEF < 35%.

## Figures and Tables

**Table 1:** Baseline characteristics.

	eGFR < 30 mL/min (*n* = 554)		eGFR 30–<60 mL/min (*n* = 2213)		eGFR ≥ 60 mL/min (*n* = 4857)		*p* Value
**Age**, median (IQR)	76	(68–82)	77	(69–82)	65	(56–75)	**0.001**
**Male sex**, *n* (%)	333	(60.1)	1235	(55.8)	3391	(69.8)	**0.001**
**Body mass index,** kg/m^2^, median (IQR)	27.8	(24.2–31.9)	27.7	(24.6–31.3)	27.2	(24.4–30.7)	**0.002**
**Cardiovascular risk factors**, *n* (%)							
Arterial hypertension	436	(78.7)	1915	(86.5)	4148	(85.4)	**0.001**
Diabetes mellitus	238	(43.0)	863	(39.0)	1193	(24.6)	**0.001**
Hyperlipidemia	156	(28.2)	695	(31.4)	1862	(38.3)	**0.001**
**Prior medical history**, *n* (%)							
Congestive heart failure	120	(21.7)	303	(13.7)	249	(5.1)	**0.001**
Pacemaker	16	(2.9)	62	(2.8)	38	(0.8)	**0.001**
COPD	38	(6.9)	121	(5.5)	138	(2.8)	**0.001**
Liver cirrhosis	9	(1.6)	33	(1.5)	46	(0.9)	0.078
Malignancy	46	(8.3)	174	(7.9)	224	(4.6)	**0.001**
Stroke	6	(1.1)	29	(1.3)	25	(0.5)	**0.002**
**Comorbidities at index hospitalization**, *n* (%)							
Acute coronary syndrome							
Unstable angina	100	(18.1)	503	(22.7)	1423	(29.3)	**0.001**
STEMI	50	(9.0)	202	(9.1)	656	(13.5)	**0.001**
NSTEMI	111	(20.0)	375	(16.9)	899	(18.5)	0.142
Atrial fibrillation	202	(36.5)	805	(36.4)	994	(20.5)	**0.001**
Atrial flutter	13	(2.3)	59	(2.7)	88	(1.8)	0.061
Acute decompensated heart failure	112	(20.2)	419	(18.9)	394	(8.1)	**0.001**
Cardiogenic shock	66	(11.9)	151	(6.8)	104	(2.1)	**0.001**
Atrioventricular block	18	(3.2)	74	(3.3)	102	(2.1)	**0.005**
Cardiopulmonary resuscitation	83	(15.0)	191	(8.6)	277	(5.7)	**0.001**
Out-of-hospital	53	(9.6)	137	(6.2)	194	(4.0)	**0.001**
In-hospital	30	(5.4)	54	(2.4)	83	(1.7)	**0.001**
Valvular heart disease	143	(25.8)	530	(23.9)	618	(12.7)	**0.001**
Stroke	11	(2.0)	76	(3.4)	201	(4.1)	**0.025**
**LVEF**, *n* (%)							
>55	150	(31.8)	781	(39.7)	2387	(54.2)	**0.001**
45–55%	95	(20.1)	442	(22.5)	997	(22.6)	
35–44%	88	(18.6)	330	(16.8)	542	(12.3)	
<35%	139	(29.4)	414	(21.0)	477	(10.8)	
Not documented	82		246		454		

COPD, chronic obstructive pulmonary disease; LVEF, left ventricular ejection fraction; IQR, interquartile range; NSTEMI, non-ST-segment elevation myocardial infarction. Level of significance *p* ≤ 0.05. Bold type indicates statistical significance.

**Table 2:** Procedural, laboratory, and follow-up data.

	eGFR < 30 mL/min (*n* = 554)		eGFR 30–<60 mL/min (*n* = 2213)		eGFR ≥ 60 mL/mi (*n* = 4857)		*p*-Value
**Coronary angiography**, *n* (%)							
No evidence of coronary artery disease	110	(19.9)	600	(27.1)	1611	(33.2)	**0.001**
One-vessel disease	104	(18.8)	400	(18.1)	1015	(20.9)	
Two-vessel disease	121	(21.8)	449	(20.3)	987	(20.3)	
Three-vessel disease	219	(39.5)	764	(34.5)	1244	(25.6)	
Right coronary artery	311	(56.1)	1126	(50.9)	2113	(43.5)	**0.001**
Left main trunk	90	(16.2)	294	(13.3)	455	(9.4)	**0.001**
Left anterior descending	354	(63.9)	1297	(58.6)	2488	(51.2)	**0.001**
Left circumflex	299	(54.0)	1030	(46.5)	1845	(38.0)	**0.001**
Ramus intermedius	92	(16.6)	277	(12.5)	476	(9.8)	**0.001**
CABG	37	(6.7)	100	(4.5)	87	(1.8)	**0.001**
Chronic total occlusion	53	(9.6)	199	(9.0)	358	(7.4)	**0.024**
**PCI**, *n* (%)	242	(43.7)	945	(42.7)	2086	(42.9)	0.916
Right coronary artery	93	(16.8)	351	(15.9)	828	(17.0)	0.462
Left main trunk	30	(5.4)	106	(4.8)	152	(3.1)	**0.001**
Left anterior descending	116	(20.9)	496	(22.4)	1091	(22.5)	0.713
Left circumflex	78	(14.1)	315	(14.2)	688	(14.2)	0.995
Ramus intermedius	14	(2.5)	39	(1.8)	83	(1.7)	0.385
CABG	10	(1.8)	24	(1.1)	20	(0.4)	**0.001**
**Sent to CABG**, *n* (%)	26	(4.7)	95	(4.3)	216	(4.4)	0.909
**Procedural data**							
Number of stents, median (IQR)	2	(1–3)	2	(1–3)	2	(1–3)	0.620
Stent length, median (IQR)	44	(24–76)	44	(24–76)	44	(24–76)	0.597
Contrast, median (IQR)	128	(74–200)	120	(72–200)	110	(70–190)	**0.003**
**Baseline laboratory values**, median (IQR)							
Sodium, mmol/L	139	137–141)	139	(138–141)	140	(138–141)	**0.001**
Potassium, mmol/L	4.3	(4.0–4.7)	4.0	(3.7–4.3)	3.9	(3.7–4.1)	**0.001**
Calcium, mmol/L	2.2	(2.1–2.3)	2.2	(2.1–2.3)	2.2	(2.1–2.3)	**0.001**
Creatinine, mg/dL	3.1	(2.3–4.6)	1.4	(1.2–1.7)	0.9	(0.8–1.0)	**0.001**
eGFR, mL/min/1.73 m^2^	21.6	(13.6–26.7)	47.8	(40.3–54.0)	79.7	(70.1–92.4)	**0.001**
Urea, mg/dL	93.6	(72.7–122.3)	51.2	(40.2–67.8)	32.5	(26.9–40.1)	**0.001**
Hemoglobin, g/dL	10.8	(9.4–12.0)	12.5	(10.9–13.9)	13.7	(12.4–14.8)	**0.001**
WBC count, x 10^9^/L	9.5	(7.4–12.7)	9.0	(7.2–11.6)	8.9	(7.1–11.1)	**0.001**
Platelet count, x 10^9^/L	212	(167–262)	229	(185–279)	240	(199–288)	**0.001**
HbA1c, %	6.2	(5.5–7.2)	6.1	(5.6–7.2)	5.7	(5.4–6.3)	**0.001**
LDL cholesterol, mg/dL	82	(61–106)	95	(72–124)	111	(84–141)	**0.001**
HDL cholesterol, mg/dL	39	(31–48)	42	(35–53)	42	(35–53)	**0.001**
Triglycerides, mg/dL	136	(101–201)	129	(97–178)	124	(92–173)	**0.001**
C-reactive protein, mg/L	56	(15–127)	31	(11–86)	21	(8–73)	**0.001**
Procalcitonin, µg/L	0.90	(0.30–4.27)	0.43	(0.15–1.93)	0.26	(0.10–1.25)	**0.001**
Albumin, g/L	29.8	(25.9–33.1)	33.3	(29.3–36.3)	35.1	(31.8–37.8)	**0.001**
INR	1.10	(1.02–1.29)	1.08	(1.02–1.22)	1.05	(1.00–1.11)	**0.001**
NT-pro BNP, pg/mL	11,261	(4395–31,255)	3287	(1237–7904)	1168	(284–3172)	**0.001**
Creatin Kinase, U/L	134	(72–353)	125	(78–250)	138	(85–312)	**0.001**
Creatin Kinase MB, U/L	39	(21–85)	31	(21–61)	31	(21–65)	**0.031**
**Medication at discharge**, *n* (%)							
ACE-inhibitor	160	(38.2)	927	(47.0)	2537	(53.8)	**0.001**
ARB	140	(33.4)	622	(31.6)	940	(19.9)	**0.001**
Beta-blocker	339	(80.9)	1513	(76.8)	3184	(67.5)	**0.001**
Aldosterone antagonist	54	(12.9)	426	(21.6)	590	(12.5)	**0.001**
ARNI	5	(1.2)	39	(2.0)	34	(0.7)	**0.001**
SGLT2-inhibitor	6	(1.4)	106	(5.4)	235	(5.0)	**0.003**
Statin	311	(74.2)	1455	(73.8)	3493	(74.1)	0.973
ASA	281	(67.1)	1163	(59.0)	3147	(66.7)	**0.001**
P2Y12-inhibitor	211	(50.4)	898	(45.6)	2260	(47.9)	0.097
OAC	133	(31.7)	820	(41.6)	1030	(21.8)	**0.001**
**Follow-up data**, median (IQR)							
Hospitalization time	10	(4–18)	8	(4–14)	6	(4–11)	**0.001**
ICU time	0	(0–0)	0	(0–0)	0	(0–0)	**0.032**
**Primary endpoint**, *n* (%)							
Heart failure, at 36 months	183	(43.7)	582	(29.5)	771	(16.3)	**0.001**
**Secondary endpoints**, *n* (%)							
Acute myocardial infarction, at 36 months	53	(12.6)	185	(9.4)	309	(6.6)	**0.001**
Coronary revascularization, at 36 months	43	(10.3)	149	(7.6)	396	(8.4)	0.165

ACE, angiotensin-converting enzyme; ARB, angiotensin receptor blocker; ARNI, angiotensin receptor neprilysin inhibitor; ASA, acetylsalicylic acid; CABG, coronary artery bypass grafting; eGFR, estimated glomerular filtration rate; HbA1c, glycated hemoglobin; HDL, high-density lipoprotein; ICU, intensive care unit; IQR, interquartile range; LDL, low-density lipoprotein; NT-pro BNP, amino terminal pro-B-type natriuretic peptide; PCI, percutaneous coronary intervention; SGLT2, sodium glucose linked transporter 2; WBC, white blood cells. Level of significance *p* ≤ 0.05. Bold type indicates statistical significance.

## Data Availability

All datasets used in this study are available, upon reasonable request, from the corresponding author.

## References

[1] Khera A.V., Kathiresan S. (2017). Genetics of coronary artery disease: Discovery, biology and clinical translation. Nat. Rev. Genet..

[2] Malakar A.K., Choudhury D., Halder B., Paul P., Uddin A., Chakraborty S. (2019). A review on coronary artery disease, its risk factors, and therapeutics. J. Cell. Physiol..

[3] Brown J.C., Gerhardt T.E., Kwon E. (2023). Risk Factors for Coronary Artery Disease.

[4] Nakamura M., Yamashita T., Yajima J., Oikawa Y., Ogasawara K., Kirigaya H., Sagara K., Koike A., Sawada H., Aizawa T. (2009). Impact of reduced renal function on prognosis in Japanese patients with coronary artery disease: A prospective cohort of Shinken Database 2007. Hypertens. Res..

[5] Manjunath G., Tighiouart H., Ibrahim H., MacLeod B., Salem D.N., Griffith J.L., Coresh J., Levey A.S., Sarnak M.J. (2003). Level of kidney function as a risk factor for atherosclerotic cardiovascular outcomes in the community. J. Am. Coll. Cardiol..

[6] Sarnak M.J., Amann K., Bangalore S., Cavalcante J.L., Charytan D.M., Craig J.C., Gill J.S., Hlatky M.A., Jardine A.G., Landmesser U. (2019). Chronic Kidney Disease and Coronary Artery Disease: JACC State-of-the-Art Review. J. Am. Coll. Cardiol..

[7] Shantouf R.S., Budoff M.J., Ahmadi N., Ghaffari A., Flores F., Gopal A., Noori N., Jing J., Kovesdy C.P., Kalantar-Zadeh K. (2010). Total and individual coronary artery calcium scores as independent predictors of mortality in hemodialysis patientes. Am. J. Nephrol..

[8] Rear R., Meier P., Bell R.M. (2014). Implications of Kidney Disease in the Cardiac Patient. Interv. Cardiol. Clin..

[9] Culleton B.F., Larson M.G., Wilson P.W.F., Evans J.C., Parfrey P.S., Levy D. (1999). Cardiovascular disease and mortality in a community-based cohort with mild renal insufficiency. Kidney Int..

[10] Luke R.G. (1998). Chronic Renal Failure—A Vasculopathic State. N. Engl. J. Med..

[11] Gansevoort R.T., Correa-Rotter R., Hemmelgarn B.R., Jafar T.H., Heerspink H.J., Mann J.F., Matsushita K., Wen C.P. (2013). Chronic kidney disease and cardiovascular risk: Epidemiology, mechanisms, and prevention. Lancet.

[12] Liao L., Aw T.Y., Kvietys P.R., Granger D.N. (1995). Oxidized LDL–Induced Microvascular Dysfunction. Arterioscler. Thromb. Vasc. Biol..

[13] Bakris G.L. (2012). Lipid Disorders in Uremia and Dialysis. Contrib. Nephrol..

[14] Krane V., Wanner C. (2011). Statins, inflammation and kidney disease. Nat. Rev. Nephrol..

[15] Schiffrin E.L., Lipman M.L., Mann J.F.E. (2007). Chronic Kidney Disease. Circulation.

[16] Pannier B., Guérin A.P., Marchais S.J., Safar M.E., London G.R.M. (2005). Stiffness of Capacitive and Conduit Arteries. Hypertension.

[17] Kestenbaum B.R., Adeney K.L., De Boer I.H., Ix J.H., Shlipak M.G., Siscovick D.S. (2009). Incidence and progression of coronary calcification in chronic kidney disease: The Multi-Ethnic Study of Atherosclerosis. Kidney Int..

[18] Budoff M.J., Rader D.J., Reilly M.P., Mohler E.R., Lash J., Yang W., Rosen L., Glenn M., Teal V., Feldman H.I. (2011). Relationship of Estimated GFR and Coronary Artery Calcification in the CRIC (Chronic Renal Insufficiency Cohort) Study. Am. J. Kidney. Dis..

[19] Bundy J.D., Chen J., Yang W., Budoff M., Go A.S., Grunwald J.E., Kallem R.R., Post W.S., Reilly M.P., Ricardo A.C. (2018). Risk factors for progression of coronary artery calcification in patients with chronic kidney disease: The CRIC study. Atherosclerosis.

[20] Byrne R.A., Rossello X., Coughlan J.J., Barbato E., Berry C., Chieffo A., Claeys M.J., Dan G., Dweck M.R., Galbrait M. (2023). 2023 ESC Guidelines for the management of acute coronary syndromes. Eur. Heart. J..

[21] Doganer Y.C., Rohrer J.E., Aydogan U., Barcin C., Cayci T., Saglam K. (2015). Association of renal function, estimated by four equations, with coronary artery disease. Int. Urol. Nephrol..

[22] Chen S., Zhou Y., Liang G., Wu W., Huang Z., Shi L., Gao Y., Gu X., Wang D. (2024). Predictive effect of estimated glomerular filtrate rate by creatinine or cystatin C on mortality in patients with coronary artery disease. Ren. Fail..

[23] Rapa S.F., Di Iorio B.R., Campiglia P., Heidland A., Marzocco S. (2019). Inflammation and Oxidative Stress in Chronic Kidney Disease—Potential Therapeutic Role of Minerals, Vitamins and Plant-Derived Metabolites. Int. J. Mol. Sci..

[24] Stenvinkel P., Carrero J.J., Axelsson J., Lindholm B., Heimbürger O., Massy Z.A. (2008). Emerging biomarkers for evaluating cardiovascular risk in the chronic kidney disease patient: How do new pieces fit into the uremic puzzle?. J. Intern. Med..

[25] Duranton F., Cohen G., De Smet R., Rodriguez M., Jankowski J., Vanholder R., Argilés A. (2012). Normal and pathologic concentrations of uremic toxins. J. Am. Soc. Nephrol..

[26] Barreto F.C., Barreto D.V., Liabeuf S., Meert N., Glorieux G., Temmar M., Choukroun G., Vanholder R., Massy Z.A. (2009). Serum indoxyl sulfate is associated with vascular disease and mortality in chronic kidney disease patients. Clin. J. Am. Soc. Nephrol..

[27] Lindner A., Charra B., Sherrard D.J., Scribner B.H. (1974). Accelerated atherosclerosis in prolonged maintenance hemodialysis. N. Engl. J. Med..

[28] Christoffersen C.B.E., Aarup A., Nielsen L.B., Pedersen T.X. (2017). ApoB and apoM—New aspects of lipoprotein biology in uremia-induced atherosclerosis. Eur. J. Pharmocol..

[29] Massy Z.A., Ivanovski O., Nguyen-Khoa T., Angulo J., Szumilak D., Mothu N., Phan O., Daudon M., Lacour B., Drüeke T. (2005). Uremia Accelerates both Atherosclerosis and Arterial Calcification in Apolipoprotein E Knockout Mice. J. Am. Soc. Nephrol..

[30] Kawtharany L., Bessueille L., Issa H., Hamade E., Zibara K., Magne D. (2022). Inflammation and Microcalcification: A Never-Ending Vicious Cycle in Atherosclerosis?. J. Vas. Res..

[31] Zoccali C., Bode-Böger S.M., Mallamaci F., Benedetto F.A., Tripepi G., Malatino L.S., Cataliotti A., Bellanuova I., Fermo I., Frolich J.C. (2001). Plasma concentration of asymmetrical dimethylarginine and mortality in patients with end-stage renal disease: A prospective study. Lancet.

[32] Mazzaferro S., Pasquali M., Taggi F., Baldinelli M. (2014). Progression of coronary artery calcification in predialysis patients with CKD: A prospective study. Kidney Int..

[33] Go A.S., Chertow G.M., Fan D., McCulloch C.E., Hsu C.Y. (2004). Chronic kidney disease and the risks of death, cardiovascular events, and hospitalization. N. Engl. J. Med..

[34] Piscitelli P., D’Errico M.M., Mirijello A., Santoliquido M., Salvatori M., Vigna C., Marchese N., Vendemiale G., Copetti M., Pontremoli R. (2022). Low GFR amplifies the association between coronary three-vessel disease and all-cause mortality. Nutr. Metab. Cardiovasc. Dis..

[35] D’Errico M.M., Mangiacotti A., Graziano D., Massa V., Piscitelli P., Vendemiale G., Viazzi F., Pontremoli R., Russo A., Marchese N. (2017). Kidney disease measures are associated with the burden of coronary atherosclerosis, independently of diabetes. Acta. Diabetol..

[36] Sarnak M.J., Levey A.S., Schoolwerth A.C., Coresh J., Culleton B., Hamm L.L., McCullough P.A., Kasiske B.L., Kelepouris E., Klag M.J. (2003). Kidney disease as a risk factor for development of cardiovascular disease: A statement from the American Heart Association Councils on Kidney in Cardiovascular Disease, High Blood Pressure Research, Clinical Cardiology, and Epidemiology and Prevention. Hypertension.

[37] Dupuis M.E., Nadeau-Fredette A.C., Madore F., Agharazii M., Goupil R. (2020). Association of Glomerular Hyperfiltration and Cardiovascular Risk in Middle-Aged Healthy Individuals. JAMA Netw. Open.

[38] Astor B.C., Levey A.S., Stevens L.A., Van Lente F., Selvin E., Coresh J. (2009). Method of glomerular filtration rate estimation affects prediction of mortality risk. J. Am. Soc. Nephrol..

[39] Donfrancesco C., Palleschi S., Palmieri L., Rossi B., Lo Noce C., Pannozzo F., Spoto B., Tripepi G., Zoccali C., Giampaoli S. (2013). Estimated glomerular filtration rate, all-cause mortality and cardiovascular diseases incidence in a low risk population: The MATISS study. PLoS ONE.

[40] Mahmoodi B.K., Matsushita K., Woodward M., Blankestijn P.J., Cirillo M., Ohkubo T., Rossing P., Sarnak M.J., Stengel B., Yamagishi K. (2012). Associations of kidney disease measures with mortality and end-stage renal disease in individuals with and without hypertension: A meta-analysis. Lancet.

[41] Fox C.S., Matsushita K., Woodward M., Bilo H.J., Chalmers J., Heerspink H.J., Lee B.J., Perkins R.M., Rossing P., Sairenchi T. (2012). Associations of kidney disease measures with mortality and end-stage renal disease in individuals with and without diabetes: A meta-analysis. Lancet.

[42] Matsushita K., van der Velde M., Astor B.C., Woodward M., Levey A.S., de Jong P.E., Coresh J., Gansevoort R.T., Consortium Kidney Disease Prognosis Consortium (2010). Association of estimated glomerular filtration rate and albuminuria with all-cause and cardiovascular mortality in general population cohorts: A collaborative meta-analysis. Lancet.

[43] Park M., Yoon E., Lim Y.H., Kim H., Choi J., Yoon H.J. (2015). Renal hyperfiltration as a novel marker of all-cause mortality. J. Am. Soc. Nephrol..

[44] Eriksen B.O., Løchen M.L., Arntzen K.A., Bertelsen G., Eilertsen B.A., von Hanno T., Herder M., Jenssen T.G., Mathisen U.D., Melsom T. (2014). Subclinical cardiovascular disease is associated with a high glomerular filtration rate in the nondiabetic general population. Kidney Int..

[45] Choi H.M., Hyun Y.Y., Lee K.B., Kim H. (2015). High estimated glomerular filtration rate is associated with coronary artery calcification in middle-aged Korean men without chronic kidney disease. Nephrol. Dial. Transplant..

[46] Spadafora L., Betti M., D’Ascenzo F., De Ferrari G., De Filippo O., Gaudio C., Collet C., Sabouret P., Agostoni P., Zivelonghi C. (2025). Impact of In-Hospital Bleeding on Post-Discharge Therapies and Prognosis in Acute Coronary Syndromes. J. Cardiovasc. Pharmacol..

[47] Hakopian N.N., Gharibian D., Nashed M.M. (2019). Prognostic Impact of Chronic Kidney Disease in Patients with Heart Failure. Perm. J..

[48] Park C.S., Park J.J., Oh I.Y., Yoon C.H., Choi D.J., Park H.A., Kang S.M., Yoo E.S., Kim J.J., Cho M.C. (2017). Relation of Renal Function with Left Ventricular Systolic Function and NT-proBNP Level and Its Prognostic Implication in Heart Failure with Preserved versus Reduced Ejection Fraction: An analysis from the Korean Heart Failure (KorHF) Registry. Korean Circ. J..

[49] Hillege H.L., Nitsch D., Pfeffer M.A., Swedberg K., McMurray J.J., Yusuf S., Granger C.B., Michelson E.L., Ostergren J., Cornel J.H. (2006). Renal function as a predictor of outcome in a broad spectrum of patients with heart failure. Circulation.

[50] McAlister F.A., Ezekowitz J., Tarantini L., Squire I., Komajda M., Bayes-Genis A., Gotsman I., Whalley G., Earle N., Poppe K.K. (2012). Renal dysfunction in patients with heart failure with preserved versus reduced ejection fraction: Impact of the new Chronic Kidney Disease-Epidemiology Collaboration Group formula. Circ. Heart. Fail..

[51] Ahmed A., Rich M.W., Sanders P.W., Perry G.J., Bakris G.L., Zile M.R., Love T.E., Aban I.B., Shlipak M.G. (2007). Chronic kidney disease associated mortality in diastolic versus systolic heart failure: A propensity matched study. Am. J. Cardiol..

[52] Damman K., Valente M.A.E., Voors A.A., O’Connor C.M., van Veldhuisen D.J., Hillege H.L. (2013). Renal impairment, worsening renal function, and outcome in patients with heart failure: An updated meta-analysis. Eur. Heart J..

[53] Schupp T., Weidner K., Lau F., Forner J., Schmitt A., Reinhardt M., Abel N., Ayasse N., Bertsch T., Akin M. (2024). Effect of severity and etiology of chronic kidney disease in patients with heart failure with mildly reduced ejection fraction. Clin. Res. Cardiol..

[54] Mielniczuk L.M., Pfeffer M.A., Lewis E.F., Blazing M.A., de Lemos J.A., Shui A., Mohanavelu S., Califf R.M., Braunwald E. (2008). Estimated glomerular filtration rate, inflammation, and cardiovascular events after an acute coronary syndrome. Am. Heart J..

[55] Anavekar N.S., McMurray J.J., Velazquez E.J., Solomon S.D., Kober L., Rouleau J.L., White H.D., Nordlander R., Maggioni A., Dickstein K. (2004). Relation between renal dysfunction and cardiovascular outcomes after myocardial infarction. N. Engl. J. Med..

[56] Yandrapalli S., Christy J., Malik A., Wats K., Harikrishnan P., Aronow W., Frishman W. (2022). Impact of Acute and Chronic Kidney Disease on Heart Failure Hospitalizations After Acute Myocardial Infarction. Am. J. Cardiol..

[57] Moukarbel G.V., Yu Z.F., Dickstein K., Hou Y.R., Wittes J.T., McMurray J.J., Pitt B., Zannad F., Pfeffer M.A., Solomon S.D. (2014). The impact of kidney function on outcomes following high risk myocardial infarction: Findings from 27 610 patients. Eur. J. Heart. Fail..

[58] Li C., Hu D., Ma C., Yang J., Song L., Shi X. (2015). The impact of admission renal dysfunction on in-hospital and long-term outcome of patients with ST-elevation myocardial infarction in Beijing. Chin. J. Intern. Med..

[59] Pasha K., Ali M.A., Habib M.A., Debnath R.C., Islam M.N. (2011). In-hospital outcome of patients with acute STEMI with impaired renal function. Mymensingh Med. J..

[60] Pavlovic A.S., Milasinovic D., Mehmedbegovic Z., Dedovic V., Jelic D., Zaharijev S., Zobenica V., Zivkovic I., Dudic J., Vukcevic V. (2019). Synergistic impact of renal failure and left ventricular dysfunction on short- and long-term mortality in patients with STEMI undergoing primary PCI. Eur. Heart J..

[61] Bernard V., El Khoury C., Fraticelli L., OSCAR Research Group (2019). Impact of renal dysfunction in patients with acute myocardial infarction on early management and outcome: A first observational French study. Eur. Heart J..

[62] Goldenberg I., Subirana I., Boyko V., Vila J., Elosua R., Permanyer-Miralda G., Ferreira-Gonzalez I., Benderly M., Guetta V., Behar S. (2010). Relation between renal function and outcomes in patients with non-ST-segment elevation acute coronary syndrome: Real-world data from the European Public Health Outcome Research and Indicators Collection Project. Arch. Intern. Med..

[63] Hanna E.B., Chen A.Y., Roe M.T., Wiviott S.D., Fox C.S., Saucedo J.F. (2011). Characteristics and in-hospital outcomes of patients with non-ST-segment elevation myocardial infarction and chronic kidney disease undergoing percutaneous coronary intervention. JACC Cardiovasc. Interv..

[64] Flores-Blanco P.J., López-Cuenca Á., Januzzi J.L., Marín F., Sánchez-Martínez M., Quintana-Giner M., Romero-Aniorte A.I., Valdés M., Manzano-Fernández S. (2016). Comparison of Risk Prediction With the CKD-EPI and MDRD Equations in Non-ST-Segment Elevation Acute Coronary Syndrome. Clin. Cardiol..

[65] Wang H.T., Chen Y.L., Wu C.J. (2016). Impact of chronic kidney disease on clinical outcomes in patients with non-ST elevation myocardial infarction receiving percutaneous coronary intervention—A five-year observational study. Int. J. Cardiol..

[66] Vavalle J.P., van Diepen S., Clare R.M., Hochman J.S., Weaver W.D., Mehta R.H., Pieper K.S., Patel M.R., Patel U.D., Armstrong P.W. (2016). Renal failure in patients with ST-segment elevation acute myocardial infarction treated with primary percutaneous coronary intervention: Predictors, clinical and angiographic features, and outcomes. Am. Heart J..

[67] Paolisso P., Bergamaschi L., Cesaro A., Gallinoro E., Gragnano F., Sardu C., Mileva N., Foà A., Armillotta M., Sansonetti A. (2023). Impact of SGLT2-inhibitors on contrast-induced acute kidney injury in diabetic patients with acute myocardial infarction with and without chronic kidney disease: Insight from SGLT2-I AMI PROTECT registry. Diabetes Res. Clin. Pract..

[68] Schupp T., Behnes M., Rusnak J., Weidner K., Ruka M., Dudda J., Schmitt A., Forner J., Egner-Walter S., Ayasse N. (2024). Predictors and Prognostic Impact of Early Acute Kidney Injury in Cardiogenic Shock: Results from a Monocentric, Prospective Registry. Cardiorenal Med..

